# Striving for Excellence Sometimes Hinders High Achievers: Performance-Approach Goals Deplete Arithmetical Performance in Students with High Working Memory Capacity

**DOI:** 10.1371/journal.pone.0137629

**Published:** 2015-09-25

**Authors:** Marie Crouzevialle, Annique Smeding, Fabrizio Butera

**Affiliations:** 1 University of Lausanne, Lausanne, Switzerland; 2 Université de Savoie, Chambéry, France; University of Lethbridge, CANADA

## Abstract

We tested whether the goal to attain normative superiority over other students, referred to as performance-approach goals, is particularly distractive for high-Working Memory Capacity (WMC) students—that is, those who are used to being high achievers. Indeed, WMC is positively related to high-order cognitive performance and academic success, a record of success that confers benefits on high-WMC as compared to low-WMC students. We tested whether such benefits may turn out to be a burden under performance-approach goal pursuit. Indeed, for high achievers, aiming to rise above others may represent an opportunity to reaffirm their positive status—a stake susceptible to trigger disruptive outcome concerns that interfere with task processing. Results revealed that with performance-approach goals—as compared to goals with no emphasis on social comparison—the higher the students’ WMC, the lower their performance at a complex arithmetic task (Experiment 1). Crucially, this pattern appeared to be driven by uncertainty regarding the chances to outclass others (Experiment 2). Moreover, an accessibility measure suggested the mediational role played by status-related concerns in the observed disruption of performance. We discuss why high-stake situations can paradoxically lead high-achievers to sub-optimally perform when high-order cognitive performance is at play.

## Introduction

Striving for success and the wish to rise above others is part of most students’ lives, since academic achievement carries with it crucial consequences such as college acceptance, graduation, parents’ pride, and access to the highest-profile jobs. However, whether this motivation—which is referred to as performance-approach goals—actually facilitates or rather endangers cognitive performance is an issue that is still widely discussed in the achievement goal literature. Indeed, although a wealth of research in the area of achievement goals has shown that performance-approach goals positively predict academic achievement [1], recent research has demonstrated that performance-approach goals have the potential to activate outcome-related concerns that interfere with task focus, and impair cognitive performance [2].

Within the frame of this mixed picture, little attention has been devoted to high achievers—that is, those students who are used to performing well and rising above others at school, two features at the heart of performance-approach goal pursuit—and how they deal with the striving for excellence. In particular, the aforementioned mixed picture suggests a puzzling riddle. On the one hand, high achievers’ record of success may help them being comfortable with the pursuit of excellence and normative superiority represented by performance-approach goals. On the other hand, however, they might be particularly concerned by maintaining their outcomes and standing, and hence more prone to activate distractive outcome-related concerns under performance-approach goal pursuit. The present research addresses the mechanisms involved in this riddle. In particular, on the basis of the literature depicting Working Memory Capacity (WMC) as a strong predictor of high-order cognitive performance and academic achievement, we investigated whether performance-approach goal-related concerns might be amplified for those students who are the most cognitively efficient in complex tasks (i.e., high-WMC students), leading them to paradoxically experience more distraction than their low-WMC counterparts.

### The Paradox of Performance-Approach Goals and Performance

In most academic settings, the salience of stakes as well as the importance to succeed constitute an indisputable reality for students. Indeed, grades, ranking and assessment processes represent a central feature of academic functioning—carrying with it information regarding competence as well as consequences for self-esteem [3–5]. In particular, the selection processes that are in place in most educational settings signal the importance to get good grades and to perform among the best students, so as to achieve at the highest level and later secure a high-profile job [6,7]. This culture of high achievement has been shown to promote the endorsement of performance-approach goals—defined as the strive for excellence through outperforming others in order to demonstrate competence [8,9]. Indeed, students recognize its pursuit as adaptive and useful to succeed in the academic system [10].

The paradox of performance-approach goals lies in two sets of results. On the one hand, performance-approach goal adoption has been consistently found to predict academic performance [11–13], as well as many positive outcomes such as self-efficacy, challenge construal, effort and competence valuation [14]. On the other hand, there is growing evidence showing that performance-approach goals tend to focus students on the importance of getting good grades and demonstrating competence, sometimes to the detriment of learning and task focus [15]. In particular, the use of unethical methods such as cheating [16,17], and of strategic behaviors such as superficial learning and rote memorization [18] constitute evidence that striving for success can jeopardize the educational function of the academic system—that is, learning and gaining knowledge. In echo with these empirical findings, Elliot and Moller [14]—when confronting the normative structures of educational systems with their learning function—underline that “when normative evaluation is the primary emphasis in a learning environment, it will evoke a host of motivational concerns (e.g., self-presentation, self-validation, self-protection)” ([14] p. 347) prone to disrupt students’ desire to focus on the task and develop abilities. Hence, strongly competitive learning environments may somehow divert students from the desire to gain knowledge by instead activating esteem-based, self-appearance concerns.

In line with this reasoning, Crouzevialle and Butera [2] provided experimental evidence that performance-approach goals have indeed the potential to paradoxically reduce cognitive performance. In particular, they found that the desire to attain normative superiority over the other students could temporarily deplete working memory, by activating outcome-related concerns that interfere with task focus and hinder complex task solving. While such a divided-attention situation generated by performance-approach goals is assumed to be applicable to the general student population, the present research proposes to test whether pressure to achieve above peers could be amplified for a specific population of students—namely those who are the most used to reach high grades and outperform others. Specifically, we build on work that has consistently depicted high Working Memory Capacity (WMC) students as those who are the most cognitively efficient when dealing with complex activities [19–21], and propose that this advantage could paradoxically turn out to be harmful under situations where both performance-approach goals and uncertainty regarding chances to successfully reach them are salient.

### High-Working Memory Capacity Students and Performance-Approach Goals

Working Memory refers to the ability to both temporarily maintain and process information during high-order cognitive task completion. This cognitive ability is limited, and research has revealed inter-individual differences in Working Memory Capacity (WMC) that denote “differences in the ability to allocate attention resources” ([22] p.126) and consistently demonstrated WMC to be a strong and powerful predictor of performance in a wide range of complex cognitive activities. To name but a few, WMC is indeed positively related to learning, language comprehension, language production, and fluid reasoning [20,21,23–25], and has also been identified as a positive predictor of performance on mathematical problem solving [19, 26]. It therefore comes as no surprise that inter-individual differences in WMC, as tested through complex span tasks [27], also positively predict academic success in educational settings [28,29]. These findings imply that higher-WMC students are most likely to be successful pupils and high-achievers in the classroom, which confers them a high status in achievement-relevant contexts.

However, could this benefit turn out to be a burden in specific situations where evaluative stakes are particularly salient? This question is prompted by the aforementioned literature showing that performance-approach goals elicit self-presentation [14] as well as normative goal-attainment concerns [2] that derive from the “instrumental importance of the outcome” ([30] p. 349) inextricably tied with performance-approach goal pursuit. We propose that such concerns may be higher in students who are used to succeed.

Why? Bearing in mind that WMC is frequently associated to academic achievement [28], high-WMC individuals are used to receiving frequent positive feedbacks regarding competence in academic contexts. Hence, since individuals “are strategic and will value those domains that are most likely to produce positive outcomes to the self” ([31] p. 132), they may have developed a strong concern with succeeding in academic settings. Following this reasoning, one can argue that achievement stakes will be perceived as higher for those students who are the most concerned with succeeding. Indeed, failure or underperformance would imply negative consequences for their self-esteem [32], while success would rather increase self-esteem and generate positive self-related emotions such as pride [31]. Consequently, we propose that the higher the WMC of students committed to the goal of outperforming others, the higher the distraction, which would result in impaired performance. Moreover, we claim that this higher distraction should stem from greater activation of goal attainment-concerns, catalyzed by evaluative situations where the opportunity to reaffirm and preserve their positive status is not guaranteed.

Interestingly for this contention, research carried out among students attending high-performing high schools indeed confirms that they are fully aware of the pressure to compete and maintain high achievement for college admission [7,33,34], and express concerns related to parents’ standards, which often implies that “anything but the highest grade is a failure” ([34] p. 505). Performance-approach goal pursuit might thus carry higher stakes—and thereby trigger more interfering thoughts—for high-performing students.

Such a hypothesis, stating that high-WMC individuals might be more vulnerable and prone to cognitive disruption than their low-WMC counterparts, might seem counter-intuitive. In particular, the Working Memory literature rather suggests that high-WMC individuals, because of their extra cognitive resources, should be immune to the deleterious consequences of distraction. However, and interestingly for our contention, the non-vulnerability of high-WMC students has already been questioned by recent empirical findings, namely those documenting the Choking Under Pressure phenomenon [35]. Indeed, experimental research that has investigated the impact of evaluative pressure on cognitive performance has consistently demonstrated that high-stake situations (that is, “situations in which the desire for high-level performance is maximal”, [36] p. 101) specifically impair performance for high-WMC individuals. In particular, they lose the advantage usually stemming from their higher capacity and perform at the same level as their low-WMC counterparts [36]. This phenomenon, which has been replicated many times [36,37,38], interestingly reinforces the rationale for our hypothesis.

## Hypotheses and Overview

We conducted two experimental studies that aimed to assess whether performance-approach goal endorsement differentially impacts cognitive performance as a function of individuals’ WMC. More specifically, we assume that the more individuals are prone to succeed at high-order cognitive tasks and academic work (i.e., the higher the WMC), the more likely they should be to activate concerns about the attainment of performance-approach goals (i.e., to outperform others) under situations where success is not certain. Indeed, as noted above, the higher the WMC, the more individuals experience success and high-ranking. They may therefore perceive evaluative situations as high-stakes opportunities to reaffirm their competences and status as compared to others—thereby increasing the importance to succeed. Such concerns about outperforming others and preserving a favorable status are likely to consume working memory resources and hence interfere with task solving.

In sum, we expect that under performance-approach goal instructions, more than under goal instructions that do not put emphasis on social comparison, the higher the WMC, the lower the cognitive performance (Hypothesis 1). In the above reasoning, we posited that the activation of performance-approach goals should engender more distractive goal-attainment concerns as WMC increases; if this analysis is correct, then we should observe two effects. First, the decrease in performance generated by performance-approach goal activation as a function of WMC should be stronger in the case of an average—i.e., disappointing for high-achievers—normative feedback than if individuals receive reassuring feedback regarding their chance to succeed better than others (Hypothesis 2). Second, pursuing performance-approach goals without being reassured about the chance to attain them might increase the accessibility of thoughts associated to status as WMC increases (Hypothesis 3); this accessibility is likely to play a mediational role in the aforementioned effect of feedback on performance (Hypothesis 4).

In two experiments, participants were asked to solve a modular arithmetic task ([2,36]) both before (baseline performance) and after (post-manipulation performance) specific goal instructions and manipulation (see the Tasks and Procedure sections). In particular, Experiment 1 was designed to test the first hypothesis and contrasted performance-approach goals with an achievement goal that does not make social comparison salient, that is, mastery-approach goals. Experiment 2 tested Hypotheses 2, 3 and 4; in particular, we manipulated uncertainty about the chances to attain the assigned performance-approach goals, by providing participants with a bogus normative feedback. Moreover, additionally to performance, we measured the accessibility of status-related thoughts so as to test their role in the expected distraction effect.

## Experiment 1

### Method

#### Ethics Statement for the two experiments

Neither medical, nor health related experimentation was performed. Both experiments were conducted at University of Lausanne, Switzerland, and followed the APA Ethical Guidelines for Research (http://www.sandplay.org/pdf/APA_Ethical_Guidelines_for_Research.pdf). Participants were contacted in public areas on campus (cafeterias, library, parks); they were informed that the study was anonymous, and were entitled to decline or withdraw to from participation. Thus, participants were requested verbal, not written consent, as this was not required in Switzerland at the time of the two studies (2010–2012). This also implies that participants were anonymous right from the beginning of the experiments, as their names were not recorded at any time. Moreover, at that time no approval was needed in Switzerland to conduct non-medical research on human subjects. As stated by the Federal Administration of the Swiss Confederation (http://www.bag.admin.ch/themen/medizin/00701/00702/07558/index.html?lang=fr), the law relating to research on human subjects (i.e., constitutional article n°118b) came into effect in January 1^st^ 2014. Given this legislation, the present research project was not submitted to any research ethics board. Following the experiment, participants were fully debriefed and invited to ask any question about the research.

### Participants

One hundred and ten students enrolled in engineering, political and social sciences, arts and humanities, law, and business curricula in two French-speaking Swiss Universities volunteered in this experiment. Fourteen participants were removed from the analyses: four did more than fourteen math errors (i.e., less than 80% of accuracy) at the math calculation part of the task measuring WMC, suggesting that the task had not been performed as was required, and four participants spent less than 5,000 ms reading the slide that contained the goal manipulation. Additionally, two participants were removed because of very short response times (less than 2,500 ms) during the modular arithmetic problems solving, whatever the problem difficulty, suggesting a lack of involvement in math calculation, and four because their accuracy score at the baseline block was below (or equal to) 50%. The final sample consisted of 96 participants, 72 female and 24 male students, with a mean age of 22.02 years (*SD* = 2.64, *Min*. = 18, *Max*. *=* 44), who were randomly assigned to one of the two experimental conditions (49 and 47 participants in mastery-approach goal and performance-approach goal conditions, respectively).

### Tasks and procedure

Upon arrival at the lab, each participant was seated in front of a computer in an individual cubicle.

#### Working memory

Participants were first introduced to a French version of the automated Operation Span task (OSPAN; [39]). This task aims to measure individual’s WMC, and requires participants to simultaneously solve easy mathematic calculations (the processing component) and retain sets of letters (the storage component). More specifically, participants are presented with series of 3 to 7 arithmetic equation-letter combinations; three series of each length are randomly presented. For each combination, they first have to check the validity of equations such as (6 * 1) + 2 = 8; immediately after their response, a single letter (e.g., F) is displayed on the screen for 1,000 ms. At the end of each series, participants are asked to recall the set of letters in the same order as presented. OSPAN scores can range from 0 to 75, and are obtained by adding the number of letters included in all the perfectly recalled sets (*M* = 42.92, *SD* = 17.26).

Additionally, and in order to prevent any trade-off between mathematic calculations and letters storage, the OSPAN classic procedure [39] emphasizes to participants that their accuracy for the equation part has to remain superior or equal to 85%. Hence, during each recall part, participants’ current percentage of accurately solved mathematic calculations was displayed in red font color in the upper right corner of the screen. Notably, a feedback was also displayed after each recall, informing participants regarding how many letters they had accurately recalled.

#### Baseline performance

Upon completion of the OSPAN task, participants were informed that they would move to the second (and unrelated) part of the experiment, which required them to solve modular arithmetic problems [36]. More specifically, written instructions informed them that they would have to judge the validity of statements such as 17 ≡ 5 (mod 6). In order to do so, participants were required to first subtract the second number from the first (i.e., here, 17–5), and then divide the intermediary result by the last (mod) number. If the final result is a whole number, the statement is true; if it is a decimal number, the statement is false. Participants were asked to mentally solve modular arithmetic problems as quickly and accurately as possible, and to give their answer (true or false) by pressing one of two keys on the keyboard. Each modular arithmetic problem appeared on the screen after a 500 ms fixation point, and remained until the participant responded. A feedback (the word “correct” or “incorrect”) was then displayed for 1,000 ms, followed by a 1,000 ms inter-trial break; the subsequent problems were then individually displayed. This procedure was the same for all modular arithmetic problems in the experiment.

After a short training, participants had to solve a first block of 24 arithmetic problems. In order to vary the problems’ difficulty, 12 problems only required a single-digit no-borrow subtraction (i.e., low-demand problems, such as 7 ≡ 2 (mod 5)), while the remaining problems required a double-digit borrow subtraction operation, (i.e., high-demand problems, such as 51 ≡ 19 (mod 4)), thereby soliciting higher working memory resources. Each problem was presented only once; presentation order within each block was randomized. This first block of problems (Phase 1) served as a baseline measure of modular arithmetic performance for each participant. In order to avoid activating any performance concerns during this first block, it was presented as a training block, for which participants were simply asked to solve the problems as quickly and accurately as possible.

#### Experimental manipulations

Participants were then informed that they would now have to solve a second block of arithmetic problems for which their performance would this time be recorded. Those in the “performance-approach goal” condition next read explicit instructions that aimed at activating performance-approach goals (validated by Darnon, Harackiewicz, Butera, Mugny and Quiamzade [40], pilot study):

*During the recorded part of the task*, *the experimenters will assess your performance*. *It is important for you to be proficient*, *to perform well and to obtain a high score*, *in order to demonstrate your competence*. *You should know that a lot of students will do this task*. *You are asked to keep in mind that you should try to distinguish yourself positively*, *that is*, *to perform better than the majority of students*. *In other words*, *what we ask you here is to show your competencies*, *your abilities*.

Participants in the “mastery-approach goal” condition read instructions designed to activate mastery-approach goals. In particular, mastery-approach goals, which refer to the desire to develop competence and acquire knowledge, appeared especially suitable for our purpose to create a condition with no emphasis on social comparison. Instructions aimed to arouse task interest and utility for everyday life, and deliberately put aside any reference to score and task performance:

*In previous research*, *we have observed that practice of the arithmetic task you are solving right now benefits to cognitive functioning and leads to a progressive improvement of mental processes*. *Hence*, *this task solving can prove to be beneficial on the long-term*. *It is however necessary that you focus your attention on calculations mastery*, *so as to quickly and accurately solve each problem*, *in order to experience these benefits*. *Try to master this task as much as you can; keep in mind its practice can be beneficial to you*.

#### Post-manipulation performance

After they had read these instructions, all participants started solving the second set of problems. Similarly to the first block, they had to solve 12 low-demand and 12 high-demand problems that were randomly presented. Finally, participants were debriefed and thanked.

### Results

We assessed the influence of our manipulations on problem performance by computing a difference score, as Crouzevialle and Butera [2] did; we thus subtracted the percentage of accuracy in Phase 1 (baseline) from the percentage of accuracy in Phase 2 (post-manipulation). Hence, the higher the difference in performance, the higher the participant’s increase in performance from Phase 1 to Phase 2.

Because our hypothesis is only applicable to problems that require a large amount of working memory resources, we selected high-demand problems to run our main analysis. Indeed, this task has been widely used by Beilock and colleagues [41], and this research has clearly shown that high-demand problems solicit a larger amount of working memory resources than low-demand problems. However, since we used the materials kindly provided by Beilock and colleagues, in which low-demand problems were included, we also run the same analysis with low-demand problems, as a control.

**Fig 1:**
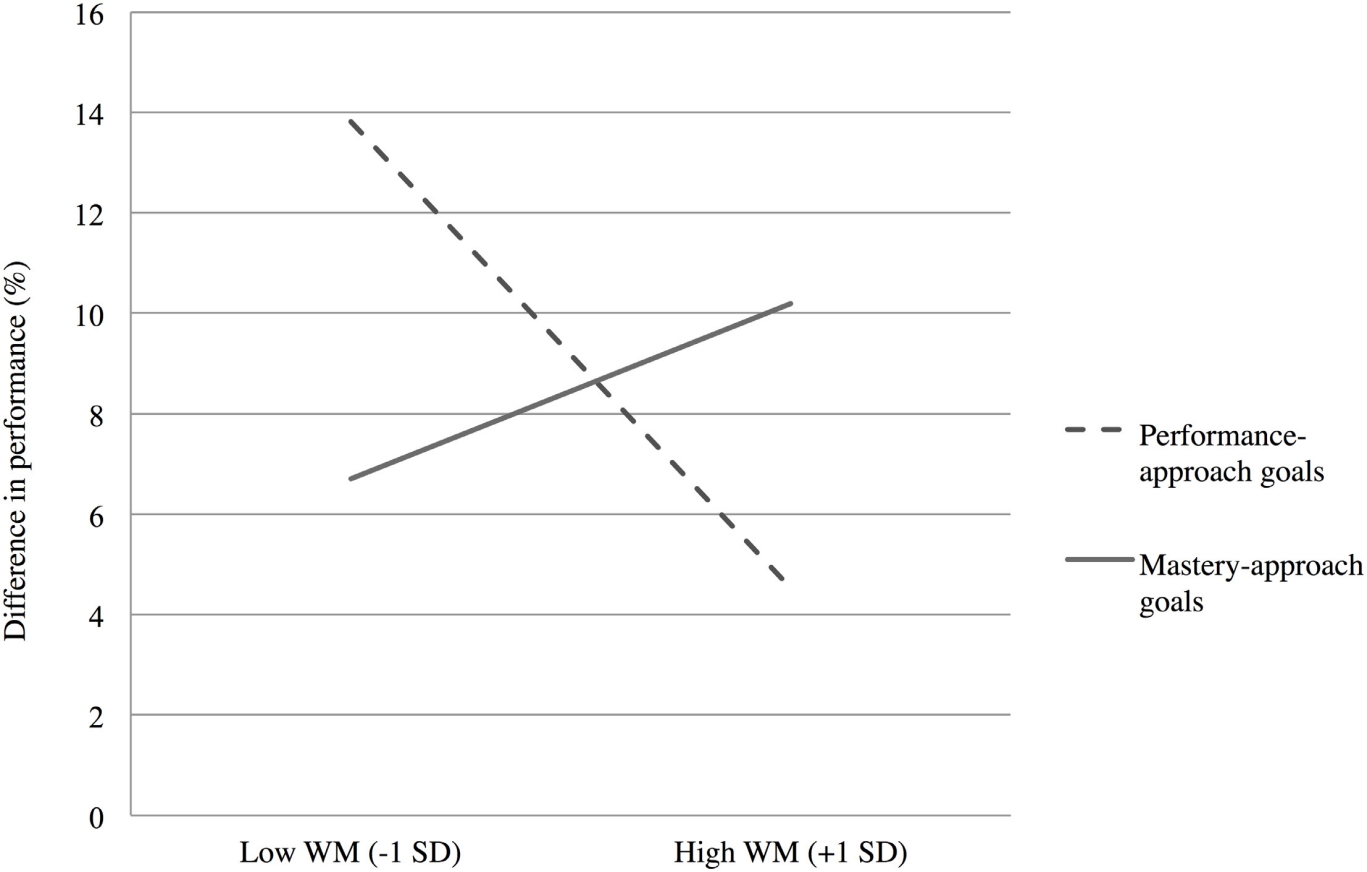
Mean difference in performance (%) for high-demand problems, as a function of experimental conditions and WMC (Experiment 1).

In order to test the influence of goal manipulation on performance as a function of WMC, we regressed the difference in performance score on WMC (mean-centered), experimental conditions (with the performance-approach goal condition coded -0.5 and the mastery-approach goal condition coded 0.5), and the interaction between WMC and experimental conditions; we additionally entered the mean-centered difference in response time (phase 2 –phase 1), as a control, as well as the interactions between this covariate and each predictor, as recommended by Yzerbyt, Muller, and Judd [42]; however, because these interactions are not theoretically relevant in the present research, they will not be discussed further. The predicted interaction between WMC and the contrast proved to be significant, *B* = 0.37, *t*(88) = 2.30, *p* < .03, *PRE* = .06. As can be seen in Fig 1, under performance-approach goal instructions, difference in performance decreased as participants’ WMC increased, a trend that did not emerge in the mastery-approach goal condition.

We also observed a significant main effect of difference in response time (phase 2 –phase 1) on difference in performance, *B* = -0.002, *t*(88) = -2.77, *p* < .01, revealing that faster response times from phase 1 to phase 2 were associated with greater performance improvement. No other effect was significant. Interested readers can consult the full report of unstandardized regression parameters and significance (for all analyses) in Tables A, B, C, D and E in S1 File. The regression analysis conducted on low-demand problems led to non-significant effects (all *t*s < 1).

It can be noted that in this article, we report the PRE (proportional reduction in error; [43]) instead of the more common eta squared. These two effect size indices are identical in their calculation and interpretation. The issue with using eta squared is that in mathematical formalization Greek letters are used to refer to population values. Eta squared should thus be the true effect size in the population, which is by definition a value that cannot be known in experimental settings. What is commonly reported in articles are estimates of eta squared in a given sample (what Judd & McClelland [43], refer to as PRE).

### Discussion

Experiment 1 sought to find evidence that motivating individuals to pursue performance-approach goals in a laboratory setting may be more interfering and detrimental to cognitive performance for high-WMC individuals than for low-WMC individuals. In particular, Hypothesis 1 predicted that the higher the WMC, the more the activation of performance-approach goals—but not the activation of mastery-approach goals—should disrupt mental calculations required during high-demand problem solving (that is, problems whose solving most heavily rely on working memory resources), thereby impairing performance. Experiment 1 findings constitute the first evidence confirming this hypothesis: As expected, under performance-approach goal instructions, the difference in performance between pre- and post-manipulation blocks decreased with the increase of participants’ WMC; this effect did not appear with mastery-approach goal instructions. Thus, Experiment 1 suggests that performance-approach goal pursuit is particularly distractive for individuals with higher WMC.

## Experiment 2

Experiment 2 sought to understand the mechanism activating the concerns supposed to underlie the observed detrimental effects of performance-approach goals in individuals with higher WMC. Hence, additionally to a condition that merely manipulated performance-approach goals, we manipulated uncertainty about the chances to attain the assigned performance-approach goals. In particular, we chose to manipulate such uncertainty by providing a bogus feedback with information about both score and ranking just before the completion of the evaluated modular arithmetic block. This bogus feedback was either very positive (high score and ranking) or average (medium score and ranking), and was designed to generate confidence vs. uncertainty regarding the chance to subsequently get a high score and outperform others. If, as we assume, high-WMC individuals activate more concerns about performance-approach goal attainment than low-WMC individuals, then being reassured about their chance to rise above others should reduce this activation, while receiving a disappointing score should maintain these distractive concerns at a high level. Thus, in order to observe the distractive effect of these goal-attainment concerns, we again measured the difference in performance at the same modular arithmetic task. Additionally, in order to gain further information regarding the nature of these concerns, we measured the activation of status-related thoughts through a lexical decision task.

### Method

#### Participants

One hundred and nineteen students volunteered in this experiment. The sample consisted of French-speaking Swiss undergraduate and graduate students enrolled in political and social sciences, arts and humanities, engineering, and business curricula. Eighteen participants were discarded from the analyses. In particular, six of them did more than fourteen math errors (i.e., less than 80% of accuracy) at the math calculation part of the OSPAN task, and four of them spent less than 5,000 ms reading the slide that contained the performance-approach goal manipulation. Additionally, six participants were discarded because of very short response times (less than 2,500 ms) during the modular arithmetic problems solving, whatever the problem difficulty, suggesting a lack of involvement in math calculation, and three because their accuracy score at the baseline block was below (or equal to) 50%. The final sample consisted of 101 participants, 65 male and 39 female students, with a mean age of 22.64 (*SD* = 5.15, *Min*. = 18, *Max*. *=* 47) who were randomly assigned to one of the three experimental conditions (32, 33, and 36 participants in performance-approach goal alone, performance-approach goal with average feedback, and performance-approach goal with positive feedback conditions, respectively).

#### Tasks and procedure

Upon arrival to the lab, participants were informed that they were about to complete different tasks that were part of the same experiment—a cover story that was important for the credibility of our manipulations, see below.

#### Baseline performance

Participants were then asked to seat in front of a computer in an individual cubicle, and were introduced to modular arithmetic problem solving through written instructions; the task was the same as that used in Experiment 1. After a short training, they had to solve a first block of 24 arithmetic problems, among which half were low-demand problems, and half were high-demand problems that solicited higher working memory resources. As in Experiment 1, this first block of problems (Phase 1) served as a baseline measure of modular arithmetic performance for each participant, and was presented as a training block, in order to avoid activating any performance concerns. Hence, participants were simply asked to solve the problems as quickly and accurately as possible.

#### Working memory

After the first block of arithmetic problems solving, participants were instructed to complete the same OSPAN task as in Experiment 1, which aimed to measure individual’s WMC; OSPAN scores ranged from 0 to 75, and were obtained by adding the number of letters included in all the perfectly recalled sets (*M* = 45.75, *SD* = 16.14). As in Experiment 1, we used the automated OSPAN task version, which provides participants—during each recall part—with their current percentage of accurately solved mathematical calculations, displayed together with a feedback regarding how many letters they have accurately recalled.

### Experimental manipulations

**Fig 2:**
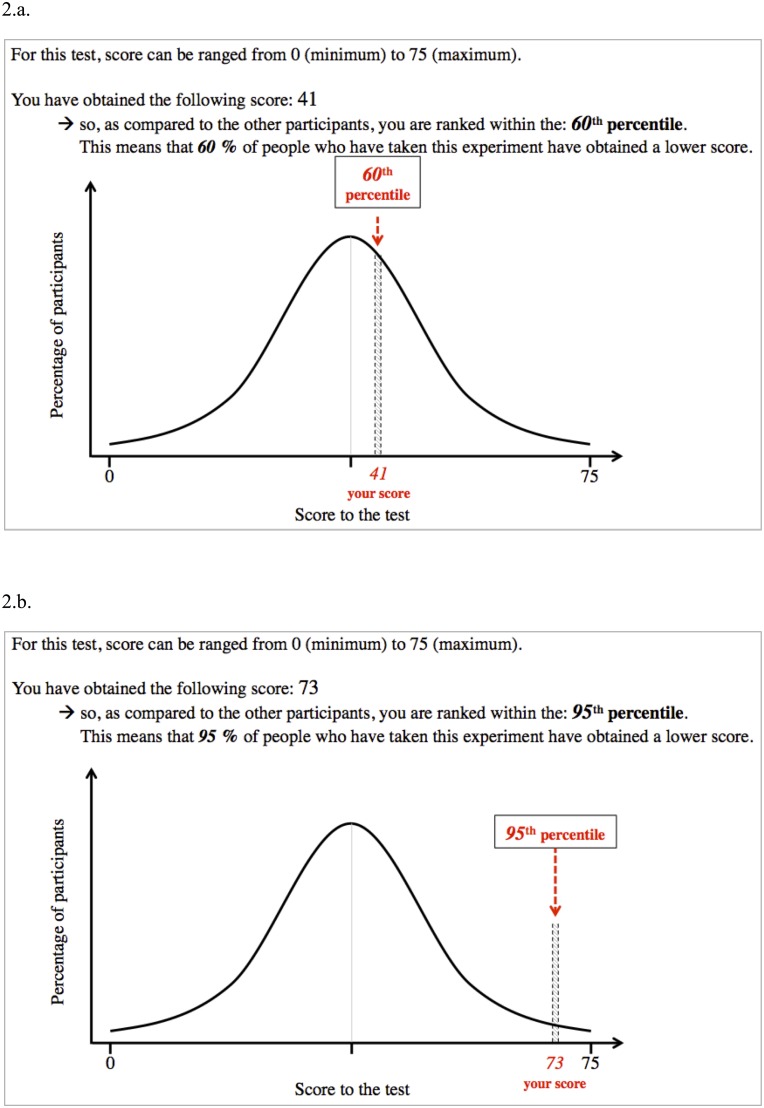
Bogus score, ranking, and normally distributed diagrams that were presented to participants of Experiment 2 in the “performance-approach goal with average feedback” (Fig 2a) and in the “performance-approach goal with positive feedback” (Fig 2b).

The manipulation occurred upon completion of the OSPAN task, and aimed at creating three conditions, namely “*performance-approach goal with average feedback*”, “*performance-approach goal with positive feedback*”, and “*performance-approach goal alone*”. Participants in the “*performance-approach goal with average feedback*” and “*performance-approach goal with positive feedback*” conditions received a bogus feedback informing them about their OSPAN score as well as the rank they had reached compared to the other participants—a feedback that was said to be delivered “for informational purposes”. Participants were told that this final score was calculated as a function of accuracy (each problem being differently weighted depending on its difficulty) and response rapidity; this complex calculation was designed to make the bogus feedback credibility difficult to challenge. In both conditions, both the bogus score and rank given to the participant were illustrated in the form of a normally distributed diagram on which the participant’s position was clearly located (cf. Fig 2a and 2b). Participants in the “*performance-approach goal with average feedback*” condition were told that they had obtained a score of 41 out of 75, leading them to be ranked at the 60^th^ percentile of the whole sample. Participants in the “*performance-approach goal with positive feedback*” condition were told that they had obtained a score of 73 out of 75, leading them to be ranked at the 95^th^ percentile of the whole sample. In order to check whether the feedback had correctly been understood, participants were then asked to recall it; none of them failed to do so. Participants in the “*performance-approach goal alone*” condition received no bogus feedback regarding their performance as compared with other participants.

#### Post-manipulation performance

Then, participants in all three conditions were informed that they would now have to solve a second block of arithmetic problems for which their performance would this time be recorded. In order to make the information carried by the previously manipulated bogus feedback relevant for this next phase, we explicitly mentioned that both tasks (the OSPAN and the modular arithmetic task) were part of the same experiment and relied on similar abilities, that is, “working memory and mental calculation”; this specification was given in each experimental condition. This explains why the OSPAN task had to be inserted between the baseline and the post-manipulation measure of the modular arithmetic task. Participants of the three conditions then read explicit instructions that aimed at activating performance-approach goals, which were similar to those used in Experiment 1. Participants next started solving the second set of problems. Similarly to the first block, they had to solve 12 low-demand and 12 high-demand problems that were randomly presented.

#### Lexical Decision Task

Upon completion of the second set, participants were introduced to the lexical decision task [44], which was presented as a verbal task designed to assess word recognition. This task involved words and non-words that were individually displayed on the center of the screen; participants were simply asked to indicate—as quickly and accurately as possible—whether the items that appeared on the screen were words or non-words, by pressing two different keys on the keyboard. This task actually aimed at measuring the accessibility of words related to status compared with filler (neutral) words (a variable we will now refer to as accessibility of status). Hence, forty items were randomly presented, among which 20 were non-words and 20 were existing French words. More specifically, 10 words were related to the concept of status (e.g., hierarchy, privilege, influence) and 10 were filler words (e.g., itinerary, lunch, nature). It is important to note that words related to status had been pilot-tested: a sample of 13 students from the same population had been asked to generate words that were related to the concept of status. Words that had been generated the most often were retained for the lexical decision task; the full list of words is available form the authors. Words were matched for frequency; non-words and words were also matched for length. Upon completion of the lexical decision task, participants were fully debriefed and thanked.

## Results and Discussion

### Overview of the Linear Regression Analyses

We first analyzed the impact of our experimental manipulations and WMC on performance, to test Hypothesis 2. Then, we tested the influence of the experimental manipulations and WMC on accessibility of status (Hypothesis 3). Finally, we assessed the mediational role of status accessibility in the interaction effect of WMC and experimental conditions on performance (Hypothesis 4).

### Difference in Performance

We again computed a difference score between the pre- and post-manipulation phases (in percentages); a positive score thus refers to an increase in performance from phase 1 to phase 2. Then, in order to test our model, we used a linear regression analysis. Because our hypothesis only deals with problems that require a large amount of working memory resources, we again selected high-demand problems to run our main analysis; however, because low-demand problems were also included in the material, we also run the same analysis with low-demand problems, as a control.

We conducted a preliminary analysis that included gender as a factor; because neither main nor interaction effects proved to be significant; this variable was not examined further. Then, in order to test our hypothesis, we created a set of two orthogonal contrasts: the first contrast testing the planned comparison was “-1–1 2”, respectively associated with the “performance-approach goal”, “performance-approach goal with average feedback” and “performance-approach goal with positive feedback” conditions; the second orthogonal contrast was “1–1 0” and meant to assess the residual variance [43]. We then regressed the difference in performance scores for high-demand problems on WMC (mean-centered), the two orthogonal contrasts, and the two interactions between each contrast and WMC; the mean-centered difference in response time for high-demand problems (Phase 2—Phase 1) was again entered as a control, as well as the interactions between this covariate and each predictor; however, because these interactions are not theoretically relevant in the present research, they will not be discussed further.

**Fig 3:**
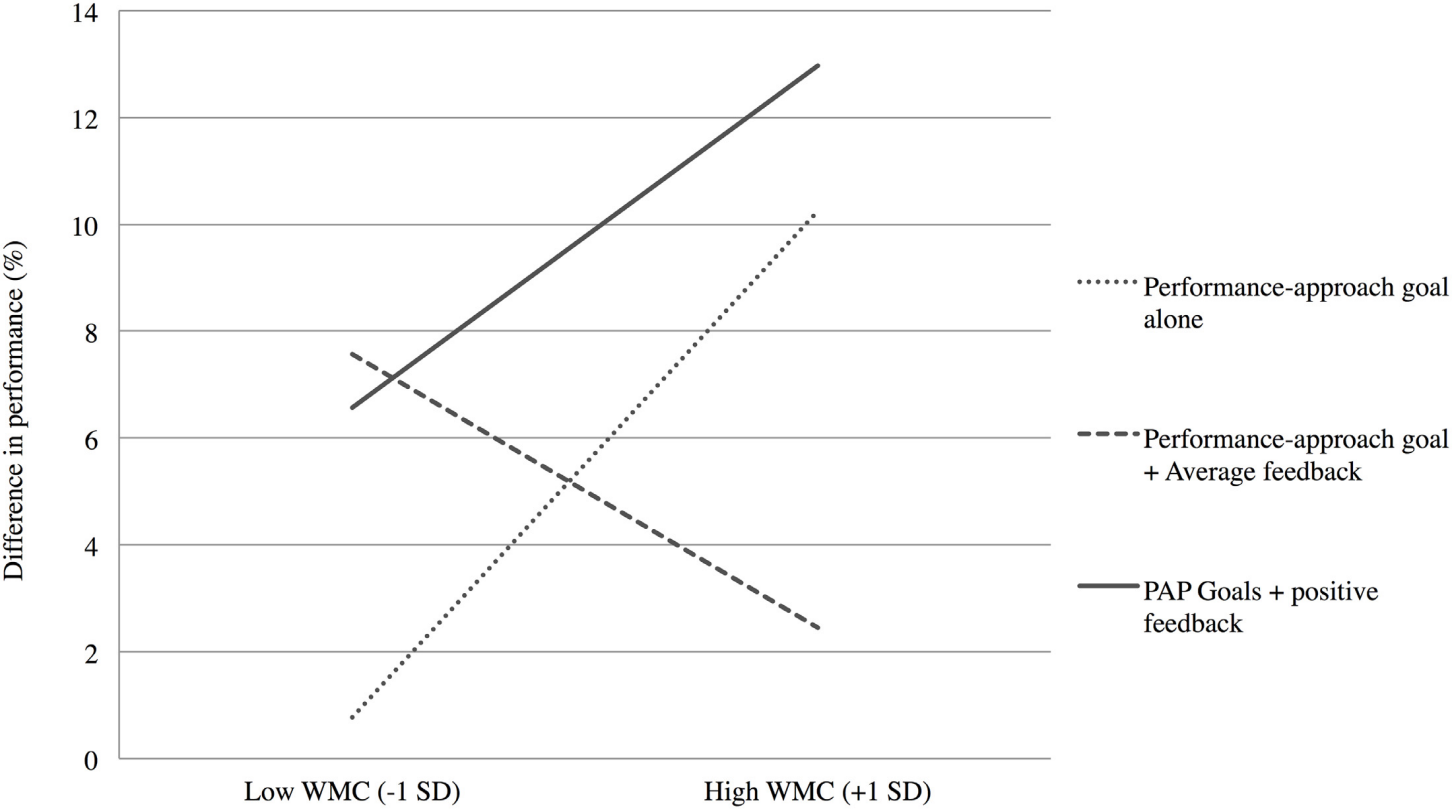
Mean difference in performance (%) for high-demand problems, as a function of experimental conditions and WMC (Experiment 2).

The interaction between WMC and the first contrast (i.e., the model) was not significant (*t* < 1), showing that our model did not fit the data well. However, WMC significantly interacted with our second contrast, *B* = 0.23, *t*(89) = 2.10, *p* < .05, *PRE* = .05; no other effect was significant. A full representation of the data is presented in Fig 3.

#### Ad interim comments

This unexpected result informs us that the overall pattern does not fit with our expectations. Indeed, the significant interaction between WMC and the orthogonal contrast indicates that the “performance-approach goal alone” condition and the “performance-approach goal with average feedback” condition performed differently as a function of participants’ cognitive capacity, and more specifically revealed a positive slope in the “performance-approach goals alone” condition—the higher the participants’ WMC, the greater their difference in performance—a result at odds with our previous finding from Experiment 1. Fig 3, however, also reveals that the pattern obtained for both the “performance-approach goal with average feedback” and “performance-approach goal with positive feedback” conditions perfectly goes in the expected direction: in line with Hypothesis 2, the lower performance generated by performance-approach goal activation as a function of WMC was observed in the case of an average—i.e., disappointing for high-achievers—feedback, but not when participants received reassuring feedback regarding their chance to succeed better than others. Could it be that the absence of replication from Experiment 1 to Experiment 2 regarding the effect of the “performance-approach goal alone” condition is attributable to some subtle modification in the setting procedure?

The main hypothesis of this research posits that high-WMC individuals, that is, individuals who are most susceptible to have accumulated achievements and high rankings in their academic life, should experience more concerns about outperforming others when assigned to pursue performance-approach goals in a laboratory setting, as compared to their low-WMC counterparts. In particular, they should view goal attainment as an opportunity to reassert their favorable position as compared to others, a representation that carries with it higher-stakes perception as well as important consequences for self-esteem. In Experiment 2, we chose to test this hypothesis by manipulating a bogus feedback designed to generate confidence versus uncertainty regarding the chance to outperform other participants. The “performance-approach goal alone” condition was intended as a control condition in which the uncertainty regarding the chance to outperform other participants (no feedback on performance) was expected to reproduce the negative relationship between WMC and difference in performance observed in Experiment 1. However, in the present procedure, was the mere “performance-approach goal” condition completely devoid of feedback regarding prior performance?

In Experiment 2, the OSPAN task—and not the first block of modular arithmetic problems as in Experiment 1—immediately preceded performance-approach goal instructions. Crucially, the OSPAN classic procedure entails that, during completion, a feedback regarding how many letters had accurately been recalled was displayed after each recall, thereby allowing participants to get an idea of whether they excelled in the task or not. Furthermore, the instructions made it very clear that the OSPAN task (which was over) and the modular arithmetic task (that they would have to complete straight after) relied on similar abilities, that is, “working memory and mental calculation”. It is therefore possible that, in the absence of any bogus feedback, this sequence gave participants the impression that (a) the task that they just completed had measured a given ability, and that (b) their performance at this task would be strongly correlated to their success at the modular arithmetic one, since both tasks relied on the same ability. This reasoning would thus imply that participants’ perceived attainability of performance-approach goals has been influenced by their OSPAN performance—a reasoning that, importantly, cannot be applied to Experiment 1, since the first block of modular arithmetic problems was inserted after the OSPAN task, and the two tasks were presented as disconnected. We could then assume that participants in the “performance-approach goal alone” condition took on the task with a high confidence regarding their chance to attain performance-approach goals—just like the participants of the “performance-approach goal with positive feedback” and unlike the participants of the “performance-approach goal with average feedback” condition. This assumption would account for the positive relationship between WMC and performance.

#### Supplementary analyses

To test this interpretation, we ran a second linear regression analysis, for which we created a new set of contrasts, the first contrast opposing the “performance-approach goal with average feedback” to both the “performance-approach goal alone” and the “performance-approach goal with positive feedback” conditions (respectively coded as “-2 1 1”), and the second contrast (“0 1–1”) testing the residual variance. Difference in performance for high-demand problems was then regressed on WMC, the two contrasts, as well as the two interactions between contrasts and WMC. The mean-centered difference in response time for high-demand problems (Phase 2—Phase 1) was also entered as a control, as well as the interactions between this covariate and each predictor; however, because these interactions are not theoretically relevant in the present research, they will not be discussed further.

Results revealed a significant interaction between WMC and the first contrast, *B* = 0.14, *t*(89) = 2.07, *p* < .05, *PRE* = .05; this finding provides evidence that the relationship between WMC and difference in performance is negative in the “performance-approach goal with average feedback” condition, and positive both in the “performance-approach goal alone” and the “performance-approach goal with high feedback” conditions. No other effect was significant. The regression analysis conducted on low-demand problems led to non-significant effects (all *t*s < 1).

Since two regression analyses are conducted on the same data, one may argue that a post-hoc correction should be applied in order to avoid Type I error. For instance, the Bonferroni correction recommends that when testing *n* hypotheses on the same data set, the significance alpha level should be adjusted at α/*n* (i.e., in our case, .05/2 = .025). This would imply that our interaction of interest, which is significant at *p* = .04, would be short of significance after the Bonferroni adjustment. However, it has been argued that this correction is very conservative and substantially reduces power—that is, the probability to accurately reject a false null hypothesis [45,46]; in other words, the Bonferroni correction lowers the probability of a type I error but increases the chance of a type II error. Moreover, in our case, the second regression analysis does not test a second hypothesis, but the focal hypothesis as it should have been formulated. Hence, we decided that a post-hoc correction was not in order and may conceal a key finding that the reconsideration of our methodological design allowed to pinpoint.

In sum, when taking the above re-interpretation into account, the second regression analysis that contrasted both the “performance-approach goals alone” and the “performance-approach goal with positive feedback” conditions with the “performance-approach goal with average feedback” condition brings support to Hypothesis 2, as it reveals a negative WMC—performance relationship only in the “performance-approach goal with average feedback” condition. Conversely, the opposite pattern appeared in both the “performance-approach goals alone” and the “performance-approach goal with positive feedback” conditions, those that provided, in different ways, reassuring information regarding the participants’ chance to outperform others: in these cases, the higher the participants’ WMC, the higher their difference in performance from pre- to post-test.

### Accessibility of Status

Eight participants were excluded from the lexical decision task analysis: one participant had to be removed due to technical problems, and seven other participants were discarded from the analysis because they were not fluent French speakers and mentioned difficulties to quickly differentiate words from non-words. Response times were log-transformed to achieve homogeneity of error variance; we however present non-transformed means, for the sake of clarity. Errors were excluded from the analysis (3.4% of the responses, 1.2% for words) as well as response latencies that were three standard deviations greater or smaller than each participant’s mean response time for words [47].

In order to test whether status accessibility was different across experimental conditions depending on participants’ WMC, we first computed a difference score for each participant, by subtracting the mean response time for status words from the mean response time for neutral words (non-words were not taken into account): the higher the score, the more accessible the status words. We then computed a set of orthogonal contrasts, as we did for our performance dependent variable. We have previously discussed the fact that the “performance-approach goal alone” condition actually contained feedback information regarding prior performance. Thus, we run a linear regression analysis testing whether the activation of status increased as WMC increased, specifically in the “performance-approach goal with average feedback” more than in the “performance-approach goal alone” and “performance-approach goal with positive feedback” conditions.

**Fig 4:**
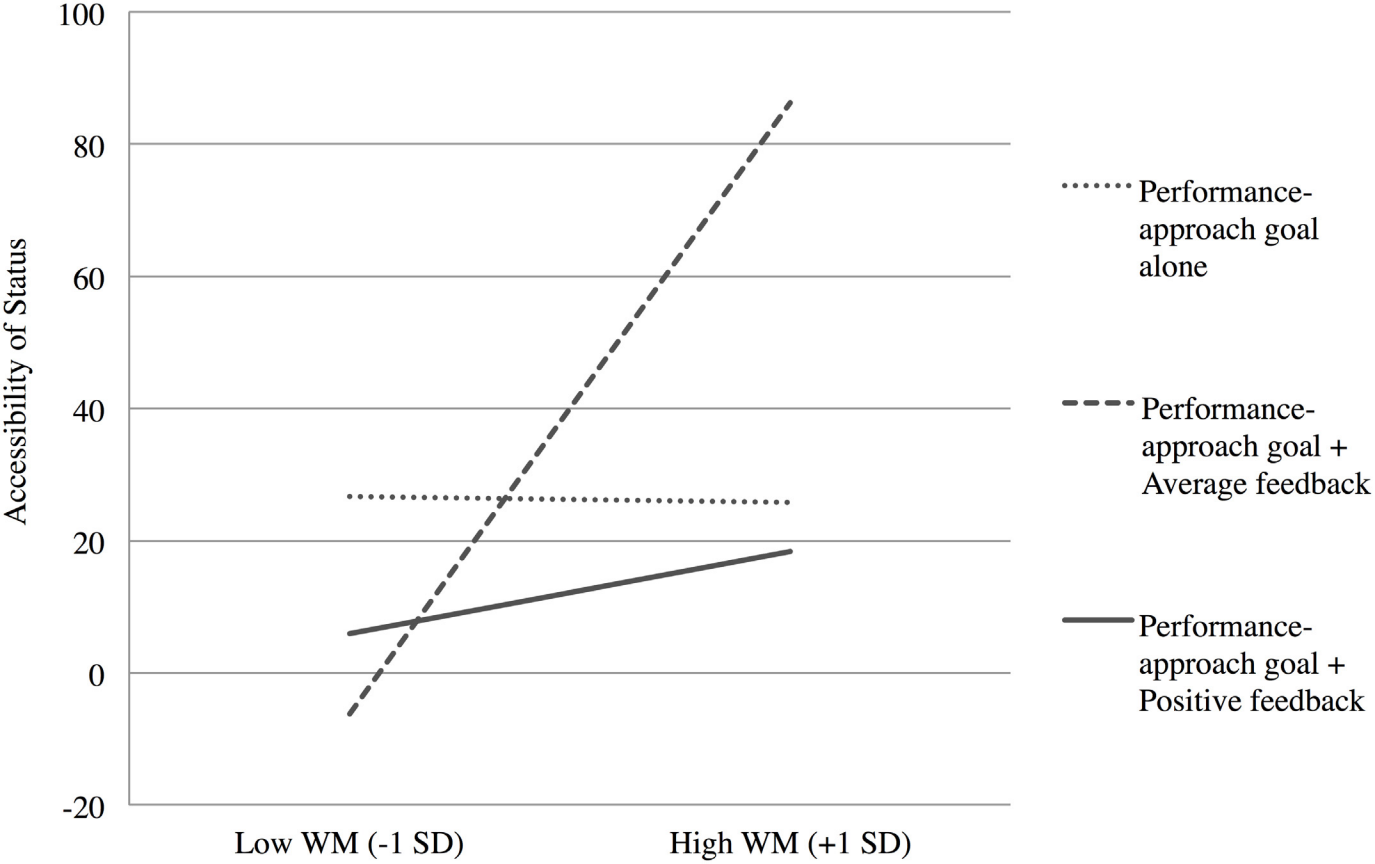
Accessibility of status as measured by the mean difference in response latencies (Neutral words—Status-related words) on the Lexical Decision Task, as a function of experimental conditions and WMC (Experiment 2).

Hence, we regressed the difference in response latencies on WMC (mean-centered), the same two orthogonal contrasts as in the second analysis presented for the difference in performance (respectively contrast 1: “-2 1 1” and contrast 2: “0 1–1”), and their interactions. Interestingly, the predicted interaction between WMC and contrast 1 appeared to be significant, *B* = -0.001, *t*(87) = -2.77, *p* < .01, *PRE* = .08: In the “performance-approach goal with average feedback” condition, the higher the WMC, the higher the activation of status-related thoughts more so than in the “performance-approach goal alone” and “performance-approach goal with positive feedback” conditions (see Fig 4). This result highlights, in line with Hypothesis 3, that the accessibility of status-related thoughts increases with WMC specifically for participants who have not been reassured about their performance, which nicely completes the result obtained with our main dependent variable, i.e., the difference in performance.

### Mediational role of accessibility of status

**Fig 5:**
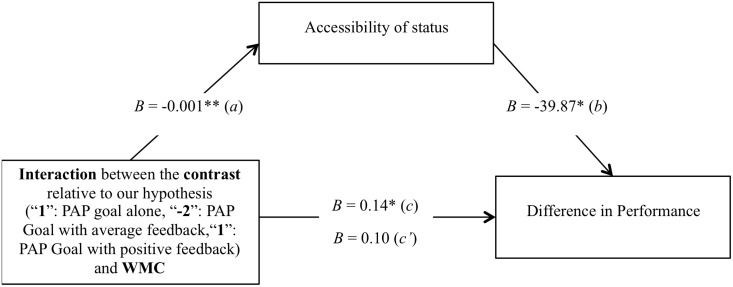
Mediation by accessibility of status of the relationship between the WMC by experimental conditions (Performance-Approach (PAP) Goal alone, PAP Goal with average feedback, PAP Goal with positive feedback) interaction and difference in performance for high-demand problems. Difference in response time for high-demand problems is controlled. All values represent unstandardized coefficients. * *p* < .05. ** *p* < .01.

To test the next and final step of our reasoning, and Hypothesis 4, we further assessed the decisive role played by status activation in participants’ arithmetic performance as a function of their WMC and of our normative feedback manipulation. In particular, we tested the mediational role of the accessibility of status (as measured through the lexical decision task) in the interaction effect between the experimental conditions and WMC on difference in performance. The summary of the results is presented graphically in Fig 5.

The conditions necessary for conducting the mediational analysis were met. In particular, as reported in the previous sections, the interaction testing Hypotheses 2 and 3—that is, the interaction between the first contrast (“1–2 1”, respectively associated with the performance-approach goals alone, performance-approach goal with average feedback, and performance-approach goal with positive feedback) and participants’ WMC—significantly predicts our main dependent variable—that is, difference in performance (*c* path). Additionally, this same interaction between the first contrast and participants’ WMC proved to be a significant predictor of the mediator variable—that is, the accessibility of status as measured with the lexical decision task (*a* path).

As a next step, we then conducted a multiple regression analysis on difference in performance for high-demand problems, with participants’ WMC (mean-centered), the model contrast, the orthogonal contrast, their interactions, and the difference in response latencies at the lexical decision task as predictors; again, the mean-centered difference in response time for high-demand problems was entered as a control, as well as the interactions between this covariate and each predictor. Supporting the mediational role of status accessibility, results showed that the higher the difference in response latencies at the lexical decision task, the lower the difference in performance, *B* = -39.87, *t*(80) = -2.08, *p* < .05, *PRE* = .05 (*b* path)—meaning that the higher the accessibility of status, the lower the difference in performance—, while the interaction between the model contrast and participants’ WMC became non-significantly related to the difference in performance, *B* = 0.10, *t*(80) = 1.47, *p* = .14 (*c’* path).

Finally, we followed the guidelines provided by Preacher and Hayes [48] and conducted a mediation analysis with 5,000 bootstrap resamples. This approach computes 95% confidence intervals associated to indirect effects; the mediation pathway is considered significant if the confidence interval does not include zero. Results confirmed that the accessibility of status-related thoughts as measured through the lexical decision task significantly mediated the relationship between the model contrast by participants’ WMC interaction and difference in performance for high-demand problems (confidence interval from 0.01 to 0.12).

## General Discussion

The present research was conducted in order to examine whether the pursuit of performance-approach goals might paradoxically endanger high-WMC individuals’ performance on cognitive tasks, to the extent that goal attainment may sometimes be uncertain. In particular, we proposed that the higher the WMC, the more students may be vulnerable to concerns about the importance of outperforming others, as attainment of performance-approach goals would imply the preservation of their high status. Since performance-approach goals have been shown to tax working memory during cognitive task completion [2], we predicted that higher WMC should result in lower cognitive performance specifically under performance-approach goal pursuit.

Firstly, and in line with this hypothesis, results from Experiment 1 confirm our hypothesis that performance-approach—as compared to mastery-approach—goals entail higher distraction during the arithmetic task solving as the students’ WMC increases. Thus, it appears that—paradoxically—it is precisely the striving for excellence that distracts higher-WMC students from the task. Secondly, Experiment 2 provides support to the hypothesis that higher-WMC students are particularly distracted by concerns with the attainment of a normatively favorable position, through the manipulation of a bogus feedback. Indeed, the negative WMC—performance relationship was stronger after the presentation of an average, disappointing feedback, than after reassuring information about performance. Such a result highlights that it is the need to outperform others without the assurance to be able to attain this goal that deprives higher-WMC students from a crucial part of their cognitive resources that cannot serve task focus, leading to performance impairment.

It is important to note that in Experiment 2, we reconsidered the meaning of the “Performance-approach goals alone” condition, and proposed that, like the “Performance-approach goal with positive feedback” condition, it provides high-WMC participants with some reassurance concerning the possible attainment of performance-approach goals. When we do that, then the results are in accordance with our Hypothesis 2 that uncertainty regarding chances to preserve a positive status constitutes an increasingly distracting element as individuals’ WMC increases.

Our third finding of interest in this research confirms the existence of these concerns with status: As shown by the result obtained with the lexical decision task in Experiment 2, the higher the WMC, the higher the accessibility of status concerns, particularly when participants pursued performance-approach goals after the presentation of an average feedback. This finding brings evidence for the first time that high-WMC individuals can activate status concerns when put into conditions of uncertainty regarding their chance to outclass others. Moreover, the mediation analysis added crucial information, as it confirmed the explanatory role played by the accessibility of status-related thoughts on the interactive effect of experimental manipulations and WMC on cognitive performance. This result supports our claim that evaluative situations where success is not guaranteed lead high-WMC individuals to activate specific status concerns, whose irrelevance regarding task focus compromises cognitive performance. It hence points at a mechanism by which high competence can turn out to be a burden when important stakes such as demonstrating higher competence and high status maintenance are at play.

From a theoretical standpoint, this research represents an important contribution to the achievement goal literature, since our findings allow shedding some light on the processes responsible for the link between performance-approach goals and achievement. Indeed, this literature has long shown that higher performance-approach goals lead to higher achievement, and that high achievers endorse performance-approach goals to a higher extent [1,14,49,50]. The existing results may therefore suggest that high achievers are comfortable with the strive for excellence represented by performance-approach goals, but the present results specify that this is the case to the extent that high-WMC students—shown to typically be high achievers [28]—are confident that they will attain their goals. Indeed, both experiments in the present study revealed that when performance-approach goals are salient and goal attainment is uncertain, WMC is paradoxically negatively related to cognitive performance, unlike situations in which these goals are not salient (Experiment 1) or the attainment of performance-approach goals seems probable (Experiment 2).

This research can additionally be related to the choking under pressure literature, as it demonstrates that the goal to outclass others carries more distractive consequences for high- than for low-WMC individuals—a result that somehow mirrors findings reported in this area [36–38]. However, even if our manipulation of performance-approach goals trigger similar cognitive mechanisms to those triggered by the choking manipulation, and both implement contexts that are meant to reproduce academic testing situations, an important difference is worth mentioning. Indeed, the high-pressure scenario used in Beilock et al.’s studies combines mentions of monetary rewards, peer pressure and social evaluation. By contrast, our performance-approach goal manipulation merely puts the emphasis on competitive social comparison and evaluation from the experimenter so as to create an evaluative context congruent with the goal priming. Notwithstanding this difference, we believe that our work may provide inspiring perspectives for research that examines the mechanisms at play under situations where one is motivated to reach high-level performance. In particular, it suggests for the first time that high-achievers may develop a perception of their level of abilities closely tied to status (and hence social comparison) considerations, which can significantly shape thought content and allocation of attention when this status is threatened. Further investigation of how individuals’ cognitive capacity and record of success influences their perception and appreciation of high-stake contexts may prove worthwhile.

This is an important endeavor as the growing salience of competition and high stakes in most educational institutions is likely to prompt feelings of pressure, and in particular pressure to perform above others [51–53]. Such competitive settings are often designed to detect and select the most brilliant students so as to guide them towards high-profile curricula and jobs. Our research nonetheless suggests two important setbacks to this belief. First, the accurate identification of individuals with the highest abilities may ironically be distorted by high-stake testing contexts, since they seemingly carry the potential of a specific cognitive disruption as WMC increases. Second, and as an important corollary, concerns that high achievers might activate under such situations can very plausibly come at the expense of interest, curiosity, and desire to acquire knowledge. Indeed, the desire to secure a favorable outcome and status may capture most of their attention and efforts and thus weaken their intrinsic motivation [54,55,56]. This possibility, which paves the way for future research, would represent a major disadvantage: It would imply that the salience of selection processes can alter the desire for learning and knowledge development among students who have the highest potential to develop expertise. Such considerations highlight the importance of further exploring the critical impact of outcome concerns and status activation [57,58] when studying the moderating role of WMC differences in the deleterious effect of evaluative contexts on performance.

## Supporting Information

S1 File Full report of unstandardized regression parameters and significance, for all analyses. Unstandardized regression parameters (*Bs*) and significance for the regression analysis including goal manipulation, WMC, the covariate and their interactions on difference in performance (Experiment 1) (**Table A**). Unstandardized regression parameters (*Bs*) and significance for the first regression analysis including WMC, the two contrasts, the covariate and their interactions on difference in performance (Experiment 2) (**Table B**). Unstandardized regression parameters (*Bs*) and significance for the second (a posteriori) regression analysis including WMC, the two contrasts, the covariate and their interactions on difference in performance (Experiment 2) (**Table C**). Unstandardized regression parameters (*Bs*) and significance for the regression analysis including WMC, the two contrasts, and their interactions on difference in response latencies (Neutral words—Status-related words) in the Lexical Decision Task (Experiment 2) (**Table D**). Unstandardized regression parameters (*Bs*) and significance for the mediation analysis studying the mediational role of status accessibility (as measured through the lexical decision task) on the relationship between the experimental conditions by WMC interaction and difference in performance (Experiment 2) (**Table E**).
(DOCX)
