# Correction: Striving for Excellence Sometimes Hinders High Achievers: Performance-Approach Goals Deplete Arithmetical Performance in Students with High Working Memory Capacity

**DOI:** 10.1371/journal.pone.0141276

**Published:** 2015-10-16

**Authors:** Marie Crouzevialle, Annique Smeding, Fabrizio Butera

The Data Availability statement for this paper is incorrect. The correct statement is: All relevant data are available from the Figshare repository: http://dx.doi.org/10.6084/m9.figshare.1537420.
