# Supplementary material for: Striving for Excellence Sometimes Hinders High Achievers: Performance-Approach Goals Deplete Arithmetical Performance in Students with High Working Memory Capacity
Source: PLoS One. 2015 Sep 25;10(9):e0137629. doi: 10.1371/journal.pone.0137629 (PMC4583442; doi:10.1371/journal.pone.0137629)
Supplement: S1 File — Unstandardized regression parameters (Bs) and significance for the regression analysis including goal manipulation, WMC, the covariate and their interactions on difference in performance (Experiment 1) (Table A). Unstandardized regression parameters (Bs) and significance for the first regression analysis including WMC, the two contrasts, the covariate and their interactions on difference in performance (Experiment 2) (Table B). Unstandardized regression parameters (Bs) and significance for the second (a posteriori) regression analysis including WMC, the two contrasts, the covariate and their interactions on difference in performance (Experiment 2) (Table C). Unstandardized regression parameters (Bs) and significance for the regression analysis including WMC, the two contrasts, and their interactions on difference in response latencies (Neutral words—Status-related words) in the Lexical Decision Task (Experiment 2) (Table D). Unstandardized regression parameters (Bs) and significance for the mediation analysis studying the mediational role of status accessibility (as measured through the lexical decision task) on the relationship between the experimental conditions by WMC interaction and difference in performance (Experiment 2) (Table E). (DOCX) [file pone.0137629.s001.docx]

**Table A. Unstandardized regression parameters (*Bs*) and significance for the regression analysis including goal manipulation, WMC, the covariate and their interactions on difference in performance (Experiment 1).**

| Variable |  |
| --- | --- |
| Goal manipulation (Performance-approach, Mastery-approach) | 0.22 |
| Working Memory Capacity (WMC) | -0.08 |
| Interaction (Goal manipulation * WMC) | 0.37 * |
| Difference in Response time (Covariate) | -2.04^-3^ ** |
| Interaction (Covariate * Goal manipulation) | -7.80^-4^ |
| Interaction (Covariate * WMC) | 0.80^-5^ |
| Interaction (Covariate * Goal manipulation * WMC) | 2.06^-5^ |

*Note. ** *p* < .03. ** *p* < .01.

**Table B.** **Unstandardized regression parameters (*Bs*) and significance for the first regression analysis including WMC, the two contrasts, the covariate and their interactions on difference in performance (Experiment 2).**

| Variable |  |
| --- | --- |
| Working Memory Capacity (WMC) | 0.14 |
| Contrast 1 | 1.46 |
| Contrast 2 | 0.20 |
| Interaction (WMC * Contrast 1) | 0.04 |
| Interaction (WMC * Contrast 2) | 0.23 * |
| Difference in Response time (Covariate) | -1.96^-5^ |
| Interaction (Covariate * WMC) | -1.13^-5^ |
| Interaction (Covariate * Contrast 1) | -8.82^-5^ |
| Interaction (Covariate * Contrast 2) | -4.03^-4^ |
| Interaction (Covariate * WMC * Contrast 1) | -8.81^-5^ ** |
| Interaction (Covariate * WMC * Contrast 2) | -3.23^-5^ |

*Note. ** *p* < .05; ** *p* < .02

**Table C.** **Unstandardized regression parameters (*Bs*) and significance for the second (a posteriori) regression analysis including WMC, the two contrasts, the covariate and their interactions on difference in performance (Experiment 2).**

| Variable |  |
| --- | --- |
| Working Memory Capacity (WMC) | 0.14 |
| Contrast 1 | 0.83 |
| Contrast 2 | -2.09 |
| Interaction (WMC * Contrast 1) | 0.14 * |
| Interaction (WMC * Contrast 2) | 0.06 |
| Difference in Response time (Covariate) | -1.96^-5^ |
| Interaction (Covariate * WMC) | -1.13^-5^ |
| Interaction (Covariate * Contrast 1) | -2.46^-4^ |
| Interaction (Covariate * Contrast 2) | -6.92^-5^ |
| Interaction (Covariate * WMC * Contrast 1) | -6.03^-5^ |
| Interaction (Covariate * WMC * Contrast 2) | 1.16^-4^ * |

*Note. ** *p* < .05.

**Table D. Unstandardized regression parameters (*Bs*) and significance for the regression analysis including WMC, the two contrasts, and their interactions on difference in response latencies (Neutral words – Status-related words) in the Lexical Decision Task (Experiment 2).**

| Variable |  |
| --- | --- |
| Working Memory Capacity (WMC) | 9.65^-4^ |
| Contrast 1 | -5.12^-3^ |
| Contrast 2 | 8.70^-3^ |
| Interaction (WMC * Contrast 1) | -1.01^-3^ ** |
| Interaction (WMC * Contrast 2) | -2.13^-5^ |

*Note.* ** *p* < .01.

**Table E. Unstandardized regression parameters (*Bs*) and significance for the mediation analysis studying the mediational role of status accessibility (as measured through the lexical decision task) on the relationship between the experimental conditions by WMC interaction and difference in performance (Experiment 2).**

| Interaction (WMC * Contrast 1) on Mediator (*a* path) | -1.01^-3^ ** |
| --- | --- |
| Direct effect of Mediator on Dependent Variable (*b* path) | -39.87 * |
| Total effect of Interaction (WMC * Contrast 1) on Dependent Variable (*c* path) | 0.14 * |
| Direct effect of Interaction (WMC * Contrast 1) on Dependent Variable (*c’* path) | 0.10 |
| Partial Effects of Control Variables on Dependent Variable: |  |
| WMC | 0.16 |
| Contrast 1 | 0.28 |
| Contrast 2 | -1.81 |
| Interaction (WMC * Contrast 2) | 0.06 |
| Difference in Response time (Covariate) | 0.3^-3^ |
| Interaction (Covariate * WMC) | -9.25^-6^ |
| Interaction (Covariate * Contrast 1) | -0.12^-2^ |
| Interaction (Covariate * Contrast 2) | -0.67^-3^ |
| Interaction (Covariate * WMC * Contrast 1) | -5.34^-5^ |
| Interaction (Covariate * WMC * Contrast 2) | 0.11^-3^ * |

*Note. ** *p* < .05. ** *p* < .01.
